# Effect of balanced versus unbalanced HES solution on cytokine response in a rat model of peritonitis

**DOI:** 10.1186/cc10858

**Published:** 2012-03-20

**Authors:** M Schläpfer, M Urner, S Voigtsberger, R Schimmer, B Beck-Schimmer

**Affiliations:** 1 University Hospital Zurich, Switzerland; 2 University of Zurich, Switzerland

## Introduction

Sepsis with multiple organ failure remains a leading cause of death in ICUs. Acute renal failure is a common complication of severe sepsis and septic shock. The effect of hydroxyethyl starch (HES) on the kidney as well as on liver tissue remains controversial and has never been tested in detail. We investigated in a model of fecal peritonitis the influence of fluid resuscitation with HES 6% in unbalanced versus balanced solutions on inflammatory mediator expression in renal and hepatic tissue.

## Methods

Cecal ligation and puncture was performed in anesthetized Wistar rats (CLP group). Sham group animals were treated in the same manner but without CLP. One hour after this procedure, Ringer lactate (RL) was intravenously infused to all animals at a volume of 30 ml/kg. Two hours after initiation of injury rats received RL (control, 75 ml/kg), unbalanced HES 130/0.42 (HES, 25 ml/kg) or balanced HES 130/0.42 (Tetraspan, 25 ml/kg). Animals were euthanized 4 hours after induction of peritonitis. Monocyte chemotactic protein-1, intercellular adhesion molecule-1, and TNFα mRNA expression were assessed in the kidneys and liver. Linear regression was used to evaluate influence of the different fluid resuscitation procedures on inflammatory mediator expression.

## Results

CLP had a significant effect on production of inflammatory mediators in the kidneys (*P *≤ 0.03) and liver (*P *≤ 0.02). While HES did not alter expression of inflammatory mediators compared to RL, fluid resuscitation with Tetraspan provoked a burst in inflammatory mediator expression, which was at least threefold higher in the kidneys (*P *< 0.001) and eightfold in the liver (*P *= 0.001) compared to the RL group.

## Conclusion

While unbalanced HES did not show a proinflammatory effect on renal and hepatic tissue in early sepsis, the balanced HES solution upregulated inflammatory mediators. Further studies have to be performed to elucidate this phenomenon in detail and to assess the functional implication of these results.

